# Is Micronucleus Assay a Suitable Biomarker for Evaluating the Cancer Risk in Professionals Exposed to Antineoplastic Drugs? A Systematic Review

**DOI:** 10.1002/jat.4973

**Published:** 2025-11-21

**Authors:** Thiago Guedes Pinto, Lorrany da Silva Avanci, Gabriel Carvalhal de Aguiar, Daniel Vitor de Souza, Patricia Ramos Cury, Ana Claudia Muniz Renno, Daniel Araki Ribeiro

**Affiliations:** ^1^ Department of Biosciences, Institute of Health and Society Federal University of São Paulo, UNIFESP Santos SP Brazil; ^2^ Department of Periodontics, School of Dentistry Federal University of Bahia Salvador BA Brazil

**Keywords:** anticancer drugs, genotoxicity, mammalian cells, micronucleus assay

## Abstract

The widespread use of antineoplastic drugs in cancer treatment has led to significant concerns regarding the potential health risks posed to healthcare professionals involved in the preparation, administration, and handling of these chemical compounds, including genotoxicity. This systematic review investigates the genotoxicity of various anticancer drugs through the micronucleus assay in mammalian cells through a comprehensive analysis of studies retrieved from PubMed, SCOPUS, and Web of Science. A systematic search conducted in May 2025 identified 28 relevant studies, all of which employed the micronucleus assay. The results indicated that 23 of the reviewed studies observed genotoxic effects linked to several drugs. As for the quality assessment, all studies (but one) were categorized as either strong or moderate; therefore, we consider our findings to be reliable. These findings raise significant concerns regarding the potential health risks associated with oncologic drugs, warranting further investigation and regulatory oversight to ensure professionals' safety. Finally, such findings are very important for clarifying the role of the micronucleus assay as a putative biomarker for evaluating the cancer risk due to anticancer drug exposure in humans.

## Abstract 2

Health professionals who are continuously exposed to antineoplastic drugs are at increased risk for cancer due to an increased incidence of micronuclei in lymphocytes and oral cells. Additionally, the micronucleus assay is an appropriate biomonitoring tool for these professionals.

## 1 Introduction

The widespread use of antineoplastic drugs in cancer treatment has led to significant concerns regarding the potential health risks posed to healthcare professionals involved in the preparation, administration, and/or handling of these agents (Roussel et al. 2019). These drugs are designed to target rapidly dividing cancer cells, but due to their nonselectivity, they can also damage normal, healthy cells, leading to harmful genotoxic and carcinogenic effects (Villarini et al. 2010; Villarini et al. 2016).

Chemotherapy agents encompass a broad range of drugs, including alkylating agents, antimetabolites, topoisomerase inhibitors, and plant alkaloids (Connor et al. 2005). These drugs vary in their mechanisms of action and genotoxic potential, which makes the evaluation of exposure risk a complex scenario. To overcome the question, occupational exposure to these drugs has been evaluated under different contexts and paradigms, demonstrating increased rates of genetic damage in healthcare professionals, as measured by different biomarkers, the micronucleus assay being the most commonly used (Ness et al. 2021; Pajic et al. 2021). This biomarker is broadly used in genotoxicity studies to assess the chromosome breakage induced by chemical substances found in the environment (Connor et al. 2005).

Micronuclei are small extranuclear bodies that form during cell division when chromosome fragments or whole chromosomes fail to incorporate into daughter nuclei, often as a result of exposure to genotoxic agents (Bonassi et al. 2011). This makes the micronucleus assay a valuable tool in biomonitoring studies, especially among individuals exposed to potentially genotoxic substances in occupational or environmental settings (Bonassi et al. 2011).

Numerous studies have shown an elevated frequency of genetic damage in healthcare professionals exposed to chemotherapy agents. Particularly, healthcare workers in hospitals have been reported to have a higher incidence of micronuclei when compared to unexposed populations (Gianfredi et al. 2017; Villarini et al. 2016). This was strongly evident in workers involved in drug preparation, such as nurses and pharmacists, where exposure levels can be significantly higher (Connor et al. 2005), potentially through inhalation of aerosols or direct dermal contact with these agents (Roussel et al. 2019). However, the data on this subject are not entirely consistent, with some studies showing no significant genetic damage in exposed workers. This inconsistency may arise from factors such as differences in study design, methodological variations, and the use of protective measures in the workplace (Gianfredi et al. 2017).

Given these challenges described above, the aim of this systematic review was to critically assess the evidence regarding the genotoxic effects through micronuclei incidence of chemotherapy drugs on healthcare workers. Furthermore, this review also seeks to evaluate the quality and reliability of the studies published on this topic. By consolidating the available evidence, this review aims to provide a clearer understanding of the occupational risks associated with chemotherapy drug exposure (Roussel et al. 2019). We understand the findings of this review will contribute to the broader understanding of the cancer risk faced by healthcare workers in oncology settings and may inspire future studies on new guidelines for improving safety protocols in these environments.

## 2 Material and Methods

This systematic review was conducted in accordance with the PRISMA 2020 guidelines and structured around the PICO model: P (Mammalian cells), I (Chemotherapeutic drugs), C (Control group), O (Genotoxicity through micronuclei formation).

Studies were deemed eligible if they fulfilled the following criteria: (1) experimental studies assessing genetic damage and/or cell death triggered by chemotherapeutic agents; (2) publications written in English; (3) data reported in compliance with internationally recognized scientific standards and suitable for extraction.

Studies were excluded based on the following criteria: (1) conference proceedings, literature reviews, editorials, or correspondence pieces; (2) full text not available in English; (3) studies with missing or nonextractable data; (4) research involving nonmammalian cell lines; (5) absence of genotoxicity assessment through the micronucleus assay; (6) unclear or incomplete outcome reporting.

### 2.1 Search Strategy

A comprehensive literature search was carried out in May 2025 using the electronic databases PubMed, SCOPUS, and Web of Science. The search strategy combined relevant descriptors using Boolean operators: (“Chemotherapeutic drug” OR “Antineoplastic agent”) AND (“Genotoxicity” OR “Mutagenicity”) AND (“Micronucleus assay”) AND (“Mammalian cells”).

To ensure thorough coverage, we also performed manual screening of reference lists from selected articles. The search was not restricted by publication date. Five independent reviewers (TGP, LSA, GCA, DVS, and DAR) conducted the initial screening of titles and abstracts. Full‐text articles were retrieved and assessed for eligibility. Any disagreements were resolved through discussion until consensus was reached. The complete search strategy is detailed in Table 1.

**TABLE 1:** Search strategy.

Electronic databases used	Search strategy (May, 2025)
PubMed https://www.ncbi.nlm.nih.gov/pubmed/Scopus https://www.scopus.com Web of Science https://www.webofscience.com/wos/alldb/basic‐search	(oncologic drugs) OR (chemotherapeutic drugs) OR (anticancer drugs) AND (DNA Damages) OR (Damage, DNA) OR (Damages, DNA) OR (DNA Injury) OR (DNA Injuries) OR (Injuries, DNA) OR (Injury, DNA) OR (Genotoxic Stress) OR (Genotoxic Stresses) OR (Stresses, Genotoxic) OR (Genotoxicity) OR (Mutagenicity) OR (micronucleus assay) (micronucleated cell) AND (mammalian) OR (mammalian cells).

### 2.2 Data Extraction and Quality Assessment

After independently extracting data from eligible studies by examining titles, abstracts, and full texts, the reviewers (TGP, LSA, GCA, DVS, and DAR) organized the relevant data following the methodology described by Guedes Pinto et al. (2024), which includes the following details: authors, publication year and country, cell types, exposure duration, assay conducted, number of cells evaluated, geno‐ and cytotoxicity assays utilized, blind analysis status, statistical methods, negative control, and key findings.

In parallel with data extraction, a risk of bias assessment was conducted using a tailored evaluation checklist adapted from previous systematic toxicology reviews by Guedes Pinto et al. (2024). The quality of the included studies was independently assessed by four reviewers (TGP, LSA, GCA, DVS, and DAR), with different relevant variables (confounders) considered in the evaluation (such as staining methods, minimum amount of evaluated cells, inclusion of blind analysis, and control groups, proper statistical analysis). In terms of the methodology for classifying the studies, those in which all variables were controlled were classified as strong. Conversely, studies where up to one confounder was not controlled were rated as moderate, and those with two or more uncontrolled confounders were considered weak at final rating, as described by Guedes Pinto et al. (2024).

## 3 Results

### 3.1 Study Selection

**FIGURE 1:**
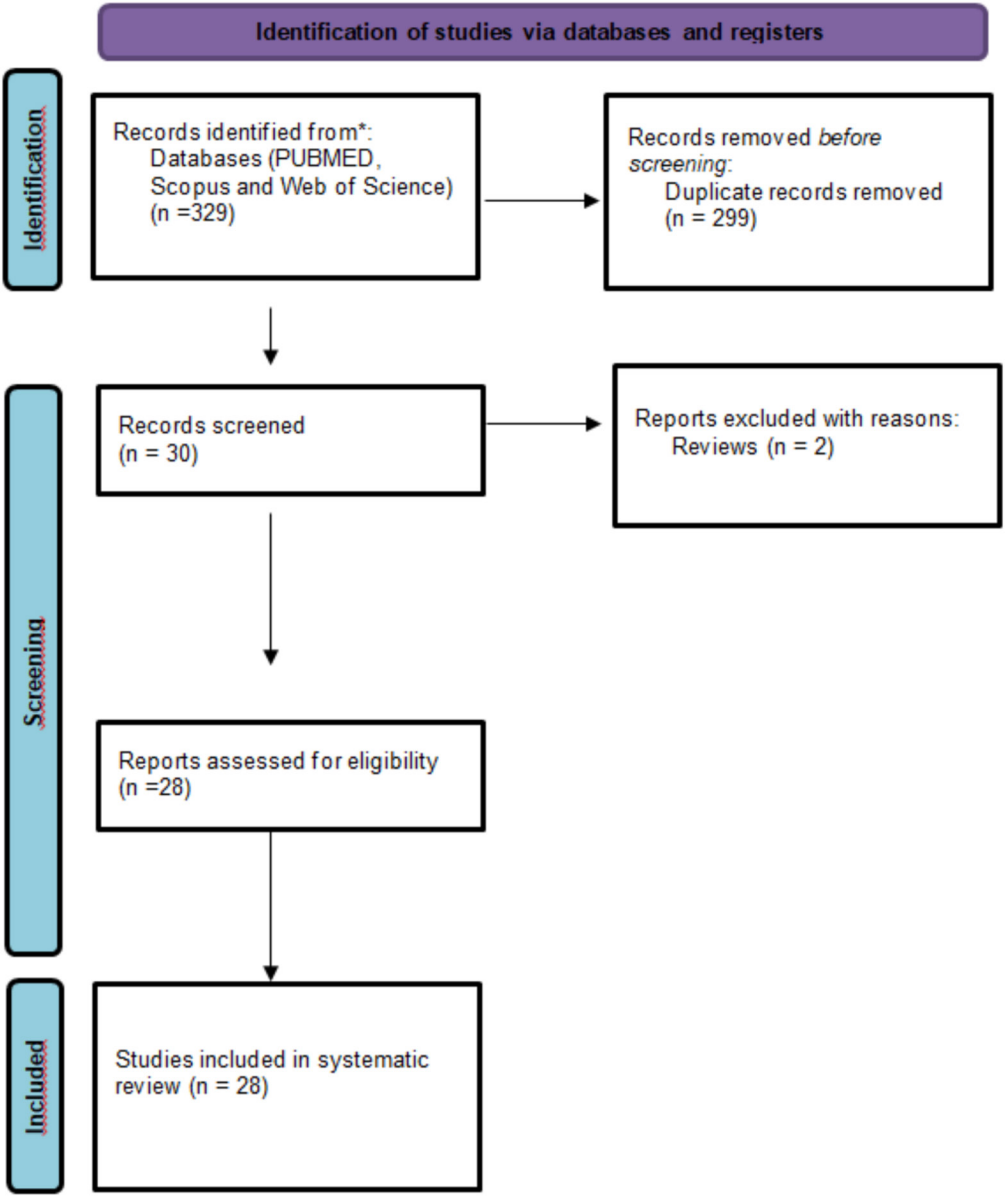
Flow chart of the study evaluating the occupational exposure to antineoplastic drugs by micronucleus assay.

The initial online search yielded 329 scientific records; however, 299 of these were duplicates and thus excluded. Following an assessment of the titles and abstracts, 2 studies were deemed irrelevant for the purposes of this research and were removed. This exclusion applied to reviews. Full manuscripts from 28 studies were carefully read by the authors of this article. The flow chart of the study is shown in Figure 1.

### 3.2 General Characteristics of the Included Studies

The most important characteristics of the studies included in the systematic review are summarized in Table 2. A total of 28 studies were evaluated, including six studies conducted in Italy, four in Brazil, three in Serbia, two in China, and the remaining studies distributed across countries such as Colombia, Egypt, Germany, India, Iran, Portugal, Spain, Tunisia, Croatia, Austria, Thailand, United Kingdom, France, Greece, and others. The types of drugs investigated were mainly antineoplastic agents, including commonly studied compounds such as cyclophosphamide, 5‐fluorouracil, bleomycin, cisplatin, doxorubicin, vincristine, methotrexate, and many others. Exposure periods ranged widely from several months to more than 20 years, with daily occupational exposure times varying between less than 1 h up to 7–8 h or more, depending on the study population and work setting. Most studies did not report exact exposure dosages. These data are detailed in Table 2.

**TABLE 2:** The most important characteristics of the studies included in the systematic review.

Author	Year of publication	Country	Type of drug	Exposure period/time	Exposure dosages
Ness et al.	2021	Brazil	Not informed	Mean: 96 months Daily occupational exposure load of at least 6 h	Not informed
Pajic et al.	2021	Serbia	Bleomycin sulfate Mitomycin Daunorubicin Actinomycine Aminouracil mustard Metotrexate Mercaptopurine Cladribin Teniposide Vinblastine Vincristine Bisulfan Chlorambucil Cisplatin Procarbazine Streptozocin Tamoxifen citrate	Daily: 1–6 h average Continually for 5–30 years	Not informed
Aristizabal‐Pachon; Castillo,	2020	Colombia	Bleomycin Carboplatin Cyclophosphamide cisplatin Doxorubicin Fluorouracil Pacli taxel	Exposure time (months): 41.10 ± 23.6 Exposure by day (hours) < 4 (*n* = 11) 27.5% and ≥ 4 (*n* = 29) 72.5% 24 individuals with less than 42 months and 16 individuals with 42 or more months.	Not informed
Santos et al.	2020	Brazil	Cyclophosphamide Ifosfamide	Hours/day: 6.43 ± 0.25 Months worked: 43.95 ± 5.78 h of daily exposure Pharmacists: 5.60 ± 1.85 Hours of daily exposure: Nurses: 7.33 ± 0.95 Months of exhibition Pharmacists: 45.41 ± 41.20 Months of exhibition– Nurses: 41.70 ± 38.96	Not informed
Ursini et al.	2019	Italy	5‐Fluorouracil Cytarabine gemcitabine Azacytidine Daunorubicin doxorubicin Epirubicin	Group exposed—Administrator antineoplastic: 15.6 ± 7.3 years Preparators antineoplastic: 18.4 ± 10.2 years	Not informed
Mahmoodi et al.	2017	Iran	Taxol Taxoter Methotrexate Cisplatin Carboplatin Adriamycin 5‐Fluorouracil Vincristine Cyclophosphamide Melphalan Actinomycin Ifosfamide Andoxal Gemzar Mebtra Velcade Busolfan Cyclosporin Oxaloplatin Vepside Doxorubicin Bleomycin sulfate	Job duties handling chemotherapy for the past 6 months	Not informed
Ladeira et al.	2014	Portugal	5‐Fluorouracil	Years of employment: 6.01 (mean ± SD, year) Range 0.17–30	Not informed
Mrđanović et al.	2012	Serbia	Cyclophosphamide Etoposide Cisplatin Doxorubicin Mitomycin c 5‐Fluorouracil Methotrexate Vincristine Carboplatin Paclitaxel	Exposure time to cytostatics: 10 years	Not informed
Villarini et al.	2012	Italy	5‐Fluorouracil Cytarabine Cyclophosphamide Gemcitabine Ifosfamide Rituximab Methotrexate Etoposide Carboplatin Dacarbazine Paclitaxel Trastuzumab Others	Job senioritya ≤10 years: 31, 11–20 years: 13 and ≥ 20 years: 4	Not informed
El‐Ebiary et al.	2011	Egypt	Cyclophosphamide Cisplatin Adriamycin Mitomycin C 5‐fluorouracil Methotrexate	Years of exposure (mean SD) = Pharmacists: 5.8 ± 3.34 Nurse: 10.3 ± 4.52	On average, 550 doses of different drug mixtures are prepared and administered every week
Bouraoui et al.	2011	Tunísia	Bleomycin Melphalan Cisplatin Busulfan Cyclophosphamide	The mean handling time was 6 h/day 55% of our exposed have duration of exposure less than 5 years and only 25% superior to 10 years.	Not informed
Kopjar et al.	2009	Croatia	Not informed	Exposed subjects handled a diversity of antineoplastic drugs. Mean exposure time for the exposed group was 12.9 years	Not informed
Rombaldi et al.	2009	Brazil	Not informed	5 days of work	Not informed
Cornetta et al.	2008	Italy	Azathioprine Busulfan Cyclophosphamide Citarabine Doxorubicin Etoposide Fluorouracil Hydroxyurea Methotrexate Thiotepa Tretinoin Vincristine	30 nonconsecutive working days	
Rekhadevi et al.	2007	India	Most frequently antineoplastic drugs: cisplatin, carboplatin, adriamycin, bleomycin, endoxane	Years of employment: 6–23 years Mean: ± 13,61 years	Not informed
Cavallo et al.	2007	Italy	Day‐care hospital nurse: Aneugens (vincristine and paclitaxel) 30–40 Alkylating agents (cyclophosphamide) 70–80 Others (5‐fluorouracil, methotrexate, epirubicin, gemcitabinea, cisplatin) Ward nurse: Aneugens (vinorelbineb, vincristine, paclitaxel, docetaxelb) Alkylating agents (cyclophosphamide, ifosfamideb) Others (etoposideb, epirubicin, cisplatin, bleomycinb, doxorubicinb, 5‐fluorouracil, cytarabinb, methotrexate, carboplatinb, ET–743b, epiadriamycinb)	Day‐care hospital nurse: Aneugens: 30–40 times at week; alkylating agents: 70–80 times at week; others: 10–60 times at week. Ward nurse: Aneugens: 3–10 times at week; alkylating agents: 1–10 times at week; others: 2–10 times at week.	Not informed
Hongping et al.	2006	China	Antineoplastic drug vincristine	Exposure years: 1–20 years	Not informed
Hongping et al.	2005	China	Cyclophosphamide, vincristine, vinblastine, cis‐platinum, 5‐fluorouracil, bleomycin, MTX, and adriamycin.	Exposure years: 1–14 years	Not informed
Cavallo et al.	2005	Italy	Cyclophosphamide 5‐Fluorouracil Ifosfamide Cytarabine Gemcitabine	Day‐care hospital nurse: 300 administration/week Ward nurse: 35 administration/week Pharmacy technicals: responsible for the preparation.	Not informed
Yang et al.	2002	China	Not informed	Nurses: average exposure duration: 5.5 years; Exposure to average 8.25 chemotherapeutic preparations daily	Not informed
Hessel et al.	2001	Germany	Cyclophosphamide Ifosfamide Doxorubicin Epirubicin Daunorubicin Idarubicin Cisplatin Carboplatin	Exposure to antineoplastic agents ranged from 1 to 144 months (mean 53 months).	Not informed
Maluf and Erdtmann	2000	Brazil	Antineoplastic drugs include alkilants (e.g., cyclophosphamide), antimetabolics (e.g., fluoruracyl), spindle poisons (e.g., vincristeine), antibiotics (e.g., doxorubicin), and hormonal therapies (e.g., diethylstilbestrol).	A follow‐up study carried out 4 years.	Not informed
Pilger et al.	2000	Austria	Bleomycin Carboplatin Cisplatin Cyclophosphamide Dacarbazine Doxorubicin Epirubicin Etoposide Fluorouracil Gemcitabine Ifosfamide Methotrexate Vincristine Vinorelbine Others cytostatic drugs in minor frequency	2 years of monitoring	Not informed
Kašuba et al.	1999	Croatia	(5‐Fluorouracil, cyclophos‐phamide, cisplatinum, methotrexate, vincristine, vinblastine).	Exposure period 1–14 years (8 nurses) Exposure period 20–31 years (12 nurses)	Not informed
Fucic et al.	1998	Croatia	Endoxan Vincristin Adriablastin 5‐Fu Alexan Bleomycin	Exposure period: 6 years, on average	Over a week: Endoxan 4000 mg Vincristin 6 mg Adriablastin 150 mg 5‐Fu 1000 mg Alexan 200 mg Bleomycin 15 mg
Kevekordes et al.	1998	Germany	Cisplatin, cyclophosphamide, iphosphamide, vincristin, doxorubicin, dactinomycin, cytosine arabinoside, and methotrexate	Exposure period: 2 months after a new safety hood was installed and 7 months later	Not informed
Machado‐Santelli et al.	1994	Brazil	Vincristine, vinblastine, aracytosine C, 5‐fluorouracyl, cyclofosfamide, cisplatin, methotrexate, bleomycin, mitomycin, and adriablastin	We considered as exposed only individuals who regularly make up dilutions of the drugs	Not informed
Anwar et al.	1994	Egypt	Cyclophosphamide, phenylalanine mustard, thiotepa, busulfan, methotrexate, 5‐fluorouracil, 6‐mercaptopurine, vinblastine, adriamycin, bleomycin, mitomycin C, and cisplatinum	Duration of exposure (months): 79.9 ± 46.2	Not informed

Table 3 provides a comprehensive summary of the primary methodological and demographic characteristics of the 28 studies included in this systematic review, having all of them conducted the micronucleus assay. The selected studies evaluated genotoxic effects of chemotherapeutic agents on mammalian cells in vivo, primarily focusing on lymphocytes from peripheral blood and buccal epithelial cells. The number of participants, gender distribution, and age ranges are reported for both control and exposed groups.

**TABLE 3:** Variables related to occupational exposure to antineoplastic drugs and genotoxicity (micronucleus assay).

Authors	Cell type	*n*	Gender	Age	No. evaluated units in the MN assay	Stain	Evaluated parameters for the MN assay	Inclusion criteria	Cito‐toxicity analysis	Blind analysis	Proper statistical description	Control group
Ness et al. 2021	Buccal epithelial cells Lymphocytes from peripheral blood	Control: Nurse: 5 Pharmacist/Pharmacytechnician: 10 Other professions: 20 Exposed: Nurse: 20 Pharmacist/Pharmacytechnician: 9 Other professions: 0	Male (12) Female (52)	Not exposed: 42.00 years Exposed: 41.00 years	1000 cells	GelRed	Cell counting	Yes	No	No	Yes (Shapiro–Wilk, Student's *t* test, Mann–Whitney test, chi‐square test)	Yes
Pajic et al. 2021	Lymphocytes from peripheral blood	Control: 201 Exposed: 222	Control: Male (99) and female (102) Exposed: Male (106) and female (116)	Control: 42.31 ± 8.02 Exposed: 40 ± 13	1000 binucleated cells	Giemsa	Cell counting	Yes	Yes (apoptotic cells count, necrotic cells count and nuclear division index)	Yes	Yes (Kolmogorov–Smirnov test, Student *t* test, chi‐square test, Mann–Whitney test, Kruskal–Wallis)	Yes
Aristizabal‐Pachon and Castillo 2020	Lymphocytes from peripheral blood	Control: 40 Exposed drug administration: 12 Pharmacy: 26 Other: 2	Control female (32) and male (8) Exposed female (32) and male (8)	Control: 30.80 ± 5.5 years Exposed: 32.05 ± 5.1 years	1000 binucleated cells	Giemsa	Cell counting	Yes	Yes (lymphocytes were isolated was assayed for viability)	No	Yes (chi‐square, Fisher's, Pearson's χ^2^, Student *t* test, Mann‐Whitney *U* test, Kruskal–Wallis)	Yes
Santos et al. 2020	Buccal epithelial cells Urine	Control: 10 Exposed: 49	Control female (5) and male (5) Exposed female (35) and male (14)	Control minimum: 21 and maximum: 50 years Exposed minimum: 24 and maximum: 57 years	1000 cells	Giemsa Ethidium bromide	Cell counting	Yes	No	No	Yes (Tukey–Kramer, Kruskal–Wallis, Dunn according)	Yes
Ursini et al. 2019	Buccal epithelial cells Urine	Control: 53 Exposed administrator: 25 and preparator: 17	Control female (13) and male (15) Exposed administrator female (20) and male (5) Preparators female (8) and male (9)	Control: 40.9 ± 11.0 years Exposed administrator: 40.0 ± 9.2 years and Preparators: 42.8 ± 9.3 years	2000 cells	Acridine Orange Gel red	Cell counting (MN, NB, CC, BE)	Yes	Yes (advanced stage of necrosis and apoptosis)	No	Yes (ANOVA, Mann–Whitney *U* test, Kruskal–Wallis)	Yes
Mahmoodi et al. 2017	Lymphocytes from peripheral blood	Control: 74 Exposed tochemo‐therapeutics: 81	Female and male	Control female 35.35 ± 7.95 and male 33.87 ± 6.58 years Exposed tochemo‐therapeutics: female 35.35 ± 7.95 and male 35.61 ± 7.5 years	500 binucleated cells	Giemsa	Cell counting	Yes	No	Yes	Yes (Student's *t* test)	Yes
Ladeira et al. 2014	Lymphocytes from peripheral blood	Control: 111 Exposed nurses: 27	Control female (54) and male (57) Exposed nurses female (5) and male (22)	Control: 34.25 ± 0.88 years Exposed: 34.89 ± 1.47 years		—	Cell counting (MN, lymphocytes)	Yes	No	Yes	Yes (Shapiro–Wilk test, Mann–Whitney test)	Yes
Mrđanović et al. 2012	Lymphocytes from peripheral blood	Control: 20 Exposed: 15	Only female	Control: 35.5 years Exposed: 38 years	1000 binucleated cells	Giemsa	Cell counting (MN and BN)	Yes	No	No	Yes (Mann–Whitey *U* test and ANOVA)	Yes
Villarini et al. 2012	Lymphocytes from peripheral blood	Control: 50 Exposed: 48	Control female (38) and male (12) Exposed nurses female (41) and male (7)	Control: 36.56 ± 11.17 years Exposed: 39.81 ± 9.56 years	1000 binucleated cells	Giemsa	Cell counting (MN and BN)	Yes	No	Yes	Yes (Mann–Whitney *U* test)	Yes
El‐Ebiary et al. 2011	Lymphocytes from peripheral blood	Control: 30 Exposed: Pharmacists: 18 Nurses: 20	Only female	Control: 30.86 ± 5.77 years Pharmacists: 31.38 ± 4.39 years Nurses: 31.1 ± 4.96 years	1000 binucleated cells	Giemsa	Cell counting	Yes	No	Yes	Yes (Student's *t* test, Pearson's correlation test)	Yes
Bouraoui et al. 2011	Lymphocytes from peripheral blood	Control: 20 Exposed: 20 nurses	Females (16) and male (4)	Control: 33.8 ± 8.37 years Exposed: 35.85 ± 8.05 years	2000 binucleated cells	Giemsa	Cell counting	Yes	No	No	Yes (Student's *t* test)	Yes
Kopjar et al. 2009	Lymphocytes from peripheral blood	Control: 50 Exposed nurses: 50	Only female	Control: 37.98 ± 78.96 years Exposed: 37.00 ± 78.87	1000 binucleated cells	Giemsa	Cell counting	Yes	No	Yes	Yes ((Mann–Whitney *U* test, ANOVA, Kruskal–Wallis)	Yes
Rombaldi et al. 2009	Lymphocytes from peripheral blood	Control: 20 Exposed: 20	Control: Male (2) and female (18) Exposed: Male (2) and female (18)	Control: 23–56 years Exposed: 21–54 years	1000 cells	Giemsa	Cell counting	Yes	No	Yes	Yes (*t* test, Tukey, Pearson correlation)	Yes
Cornetta et al. 2008	Lymphocytes from peripheral blood	Control: 73 Exposed: 83	Control: Male (20) and female (53) Exposed: Male (16) and female (64)	Control: 23–56 years Exposed: 26–58 years	1000 cells	Giemsa	Cell counting	Yes	No	Yes	Yes (Mann–Whitney *U* test, *t* test and multiple linear regression)	Yes
Rekhadevi et al. 2007	Lymphocytes from peripheral blood Buccal epithelial cells	Control: 60 Exposed: 60	Only female	Control: 37.95 ± 5.64 years Exposed: 38.21 ± 5.61 years	500 binucleated cells (lymphocytes) 1000 cells (buccal epithelial cells)	Giemsa (MN lymphocytes) DAPI (MN buccal epithelial cells)	Cell counting	Yes	No	Yes	Yes (Student's *t* test, multiple linear regression)	Yes
Cavallo et al. 2007	Lymphocytes from peripheral blood Buccal epithelial cells	Control: 20 Exposed: Day‐care hospital nurse: 10 Ward nurse: 13	Control: Male (3) and females (17) Exposed: Day‐care hospital nurse: Male (2) and females (8) Ward nurse: Male (2) and females (11)	Control: 35.5 ± 8.6 years Day‐care hospital nurse mean age: 37.6 ± 6.1 years Ward nurses: mean age, 32.7 ± 7.7 years	1000 binucleated cells (MN Lymphocytes) 2000 cells (MN buccal epithelial cells) 110 binucleated cells (FISH MN)	Giemsa (lymphocytes) Acridine Orange (buccal epithelial cells) DAPI (FISH)	Cell counting (MN and FISH)	Yes	No	Yes	Yes (one‐way ANOVA, chi‐square test, Student *t* test, Mann–Whitney *U* test)	Yes
Hongping et al. 2006	Lymphocytes from peripheral blood	Control: 15 Exposed: 15	Female (9) and male (6)	44.17 ± 2.40 years (male) 43.33 ± 1.14 years (female)	1000 binucleated cells	Giemsa	Cell counting	Yes	Yes (proliferation index)	No	Yes (Wilcoxon's rank sum test and Kendall's test)	Yes
Hongping et al. 2005	Lymphocytes from peripheral blood	Control: 21 Exposed: 21	Control female (10) and male (11) Exposed female (10) and male (11)	Control: 21–55 years Exposed: 19–50 years	1000 binucleated cells (MN)	Giemsa	Cell counting	Yes	Yes (lymphocytes were isolated was assayed for viability)	No	Yes (Student *t* test, Wilcoxon's rank sum test, Kendall's test)	Yes
Cavallo et al. 2005	Lymphocytes from peripheral blood Buccal epithelial cells	Control: 20 Exposed: Day‐care hospital nurse: 10 Ward nurse: 13 Pharmacy technician: 5	Control: Male (5) and females (25) Exposed: Day‐care hospital nurse: Male (2) and females (10) Ward nurse: Male (2) and females (11) Pharmacy technicians: Male (3) and female (2)	Control: 34.9 ± 8.5 Exposed: Day‐care hospital nurse: 37.6 ± 5.5 Ward nurse: 32.7 ± 7.7 Pharmacy technicians: 35.8 ± 9.9	1000 binucleated cells (MN lymphocytes) 2000 cells (MN buccal epithelial cells)	Giemsa (MN Lymphocytes) Acridine Orange (MN buccal epithelial cells)	Cell counting	Yes	No	No	Yes (one‐way ANOVA, χ ^2^ test)	Yes
Yang et al. 2002	Lymphocytes from peripheral blood	Control: 16 Exposed: 16	Only female	Control: Not informed Exposed: Nurses average age of 29 years;	1000 binucleated cells	Giemsa	Cell counting	Yes	No	No	Yes (Rank sum test)	Yes
Hessel et al. 2001	Lymphocytes from peripheral blood	Control: 60 Exposed: 93	Control: Male (19) and female (41) Exposed: Male (12) and female (81)	37.2 ± 10.4 (control) 36.5 ± 8.9 (exposed)	1000 cells	Giemsa	Cell counting	Yes	No	Yes	Yes (Spearman's rank correlation)	Yes
Maluf and Erdtmann 2000	Lymphocytes from peripheral blood	Control: Control: 4 years before (10) Control 4 years after (12) Exposed: Nurses 4 years before (10) Nurses 4 years after (12)	Not informed, but the sex was matched.	Control: Control: 4 years before (31.5 ± 5.38) Control 4 years after (34.75 ± 5.42) Exposed: Nurses 4 years before (29.8 ± 7.79) Nurses 4 years after (34.42 ± 4.48)	2000 binucleated cells	Giemsa	Cell counting	Yes	No	No	Yes (Mann–Whitney *U* test, Spearman rank test)	Yes
Pilger et al. 2000	Lymphocytes from peripheral blood	Control: 39 Exposed: 39	Male and female	24–54 years	2000 cells	DAPI	Cell courting	Yes	No	Yes	Yes (ANOVA)	Yes
Kašuba et al. 1999	Lymphocytes from peripheral blood	Control: Control for micronucleus assay: 16 Control for SCE assay: 20 Exposed: 20	Not informed	Control: Control for micronucleus assay: 21–50 years old Exposed: Nurses (21–50 years old)	1000 binucleated cells	Giemsa	Cell counting	Yes	No	No	Yes (chi‐square test)	Yes
Fucic et al. 1998	Lymphocytes from peripheral blood	Control: 40 Exposed: 38	Control: Only female Exposed: Not informed	Control: Not informed Exposed: 21–45 years old	500 cells	Not informed	Cell counting	Yes	Yes (mitotic activity)	Yes	Yes (test of proportions, Kolmogorov–Smirnov test)	Yes
Kevekordes et al. 1998	Lymphocytes from peripheral blood	Control: 10 Exposed: 10	Only female	Control: Not described Exposed: 23 ± 38 years	1000 binucleated cells (CBMN)	Giemsa	Cell counting (MN)	Yes	No	No	Yes (Wilcoxon, and Mann–Whitney *U* test)	Yes
Machado‐Santelli et al. 1994	Buccal ephithelial cells	Control: 25 Exposed: 25	Female and male	Control: 34.60 ± 1.54 years Exposed: 32.08 ± 1.29 years	1000 cells	Feulgen fast green	Cell counting	Yes	No	Yes	Yes (chi‐square test and Student's *t* test)	Yes
Anwar et al. 1994	Lymphocytes from peripheral blood	Control: 20 Exposed: 20	Female	Control: 28.5 ± 5.25 years Exposed: 29.6 ± 5.4 years	1000 binucleated cells	Giemsa	Cell counting	Yes	No	Yes	Yes (chi‐square test)	Yes

Abbreviations: — = not described; CC = condensed chromatin; CYP = cytochrome P450; DNMT = DNA methyltransferase; GSTM1= glutathione S‐transferase Mu 1; MN = micronucleus assay; N/A = not applicable; NB = nuclear buds; PCR‐RFLP = real‐time reverse transcription polymerase chain reaction–restriction fragment reverse; XRCC1 = x–ray repair cross complementing family.

The number of evaluated units (e.g., binucleated cells or metaphases), staining techniques (such as Giemsa, Ethidium Bromide, DAPI, or Acridine Orange), and types of endpoints assessed (e.g., micronucleus frequency) are specified for each study. Among the studies that performed the micronucleus assay in peripheral blood, only 2 evaluated less than 1000 cells, which was considered a confounder as per the methodological standard for ensuring reliable quantification of nuclear abnormalities. As for the ones that conducted the assay in oral cells, two was also the number of studies that failed to analyze the minimum number of required cells (2000 in oral cells).

The inclusion of cytotoxicity analysis and whether studies performed blind evaluation or presented a proper statistical description were also considered, along with the use of control groups. Most studies included a negative control and applied robust statistical analyses (e.g., Student's *t* test, Mann–Whitney *U* test, ANOVA, Kruskal–Wallis), although blind assessments were less frequently reported. This heterogeneity in methodological quality and design highlights the need for cautious interpretation of genotoxicity outcomes across studies.

### 3.3 Main Results

Of the 28 studies reviewed, 23 reported genotoxic effects associated with occupational exposure to antineoplastic drugs, as detected by the micronucleus assay. All studies assessed peripheral blood lymphocytes, while only three also evaluated oral mucosal cells. In these three studies, an increased frequency of micronuclei was consistently observed in oral cells, even in cases where no significant changes were detected in lymphocytes. In contrast, five studies found no statistically significant genotoxic differences between exposed and control groups in either cell type.

Cytotoxicity was less frequently assessed, with only five studies providing relevant data. Among them, some reported altered cell viability, enzymatic activity (e.g., catalase and glutathione peroxidase), or mitotic delay. However, these findings were less consistent than genotoxic outcomes, highlighting a gap in cytotoxicity assessment.

Among the few studies that analyzed genetic polymorphisms, such as XRCC1, XRCC3, and hOGG1, no statistically significant associations were found with DNA damage, although some studies reported higher genotoxic effects in females. Importantly, Pilger et al. (2000) distinguished accidental from routine exposure, showing that accidental handling of chemotherapeutic agents can result in elevated genotoxic markers, even when routine exposure levels appear safe.

Overall, these findings reinforce the genotoxic potential of occupational exposure to antineoplastic drugs, particularly through increased micronucleus frequency. The limited assessment of cytotoxicity and polymorphisms points to the need for broader, integrative biomonitoring protocols in healthcare professionals exposed to such compounds (see Table 4).

**TABLE 4:** Main findings of studies in chronological order of authors.

Authors	Genotoxicity	Observations
Ness et al. 2021	No significant difference	The study population was composed of 81.3% of women. The exposed group used PPE.
Pajic et al. 2021	⬆ MN ⬆NBUD Lymphocytes	Higher MN frequencies were associated with female sex and older age, but smoking did not influence DNA damage. Cytostasis and cytotoxicity parameters showed no variation related to exposure, sex, age, or smoking. Strong correlation between MN and NBUD. Exposure length dependent.
Aristizabal‐Pachon and Castillo 2020	⬆ MN Lymphocytes	No significant differences were found related to age and gender in the two groups. However related to gender, we found a significant increase of DNA damage in females when compared to males in exposed individuals.
Santos et al. 2020	⬆ MN Lymphocytes	—
Ursini et al. 2019	⬆ MN Lymphocytes	—
Mahmoodi et al. 2017	⬆ MN Lymphocytes	—
Ladeira et al. 2014	⬆ MN Lymphocytes	Hospital A showed a higher percentage of contaminated samples (50%) than Hospital B (8.57%), but no difference was found between contamination levels. (5‐fluorouracil).
Mrđanović et al. 2012	⬆ MN Lymphocytes AO supplementation: micronucleus frequency and was significantly reduced	The group exposed subjects was taking antioxidative supplementation by oral administration of one capsule per day of Oligogal Se.
Villarini et al. 2012	No significant differences	Mo correlations were found between job seniority, age, smoking habits and MN rates.
El‐Ebiary et al. 2011	No significant differences	Statistical analysis detected a significant difference in years of exposure between nurses and pharmacists.
Bouraoui et al. 2011	⬆ MN Lymphocytes	—
Kopjar et al. 2009	⬆ MN Lymphocytes	We also noticed that age significantly contributed to the increase of comet tail length among nonsmokers.
Rombaldi et al. 2009	⬆ MN Lymphocytes	Within the experimental group, no difference was observed between participants with different exposure times prior to the experiment, but rather between participants of younger and older ages.
Cornetta et al. 2008	⬆MN Lymphocytes	When gender is taken into account, females show a significant higher MN value when compared to males in control and exposed group.
Rekhadevi et al. 2007	⬆ MN Oral cells and lymphocytes	
Cavallo et al. 2007	⬆ MN Exfoliated buccal cells	Significant association between vinorelbine exposure and presence of centromeric signal (FISH MN+). The higher percentage of small MN associated with exposure to alkylating agents confirms the clastogenic activity of these drugs. Administration of antineoplastic drugs in wards was associated with increased FISH MN + frequency.
Hongping et al. 2006	⬆ MN Lymphocytes	
Hongping et al. 2005	⬆ MN Lymphocytes	Correlation between exposure years and micronucleus formation
Cavallo et al. 2005	⬆ MN Buccal cell	Detection of α‐fluoro‐β‐alanine exclusively in exposed nurses highlights occupational absorption. Increased MN frequency in buccal cells but not in lymphocytes may reflect local exposure (oral cavity). High contamination in administration areas reinforces occupational risk.
Yang et al. 2002	⬆ MN Lymphocytes	—
Hessel et al. 2001	No significant changes	The only monitoring consists of serum levels of platinum (a main component of cytostatic drugs) and anthracyclines, but it is important to note that serum levels of platinum and anthracyclines were not elevated in this case.
Maluf and Erdtmann 2000	⬆ MN Lymphocytes	No associations were found with age, sex, or smoking habits.
Pilger et al. 2000	No significant differences	Positive results were observed only for accidental exposure to the drugs among pharmacy personnel. However, the exposure doses are not measured.
Kašuba et al. 1999	⬆ MN Lymphocytes	A significant increase in micronucleated lymphocytes was observed in nurses exposed to cytostatic drugs with longer exposure periods, but not significant when compared to shorter exposure period.
Fucic et al. 1998	⬆ MN Lymphocytes	20 subjects had an insufficient number of cells for mitotic activity analysis.
Kevekordes et al. 1998	⬆ MN Lymphocytes	
Machado‐Santelli et al. 1994	⬆ MN Lymphocytes	Smokers and nonsmokers do not differ significantly with respect to the incidence of micronuclei.
Anwar et al. 1994	⬆ MN Lymphocytes	

Abbreviations: ⬆ MN = increased incidence of micronuclei; AO = antioxidative defense system; GSTM1: glutathione S‐transferase Mu 1; CYP = cytochrome P450; DNMT = DNA methyltransferase; NBUD = nuclear bud; PPE = individual protection equipment; XRCC1 = x–ray repair cross complementing family.

### 3.4 Quality Assessment

The methodological quality of the 28 studies included in this review indicates a generally solid foundation for the evidence presented. Almost all studies were rated as either strong or moderate, reflecting acceptable to high methodological rigor. Specifically, 12 of the studies received a strong rating, while 15 were classified as moderate and 1 was classified as weak. These studies commonly employed blind analysis, reported cytotoxicity alongside genotoxicity, and considered relevant confounding factors such as sex, age, and smoking status. Such methodological features enhance the reliability of their findings.

In addition, it is important to highlight that the observed grading assessment supports the consistency of the observed associations between occupational exposure to antineoplastic drugs and genotoxic outcomes. This quality profile reinforces the strength of the evidence base for this systematic review (see Table 5).

**TABLE 5:** Quality assessment of studies investigating the micronucleus in individuals occupationally exposed to antineoplastic drugs.

Author	No. confounders	Detail	Rating
Ness et al. 2021	1	Blind analysis	Moderate
Pajic et al. 2021	0	—	Strong
Aristizabal‐Pachon and Castillo 2020	1	Blind analysis	Moderate
Santos et al. 2020	1	Blind analysis	Moderate
Ursini et al. 2019	1	Blind analysis	Moderate
Mahmoodi et al. 2017	1	Amount of evaluated cells	Moderate
Ladeira et al. 2014	0	—	Strong
Mrđanović et al. 2012	0	—	Strong
Villarini et al. 2012	0	—	Strong
El‐Ebiary et al. 2011	0	—	Strong
Bouraoui et al. 2011	1	Blind analysis	Moderate
Kopjar et al. 2009	0	—	Strong
Rombaldi et al. 2009	0	—	Strong
Cornetta et al. 2008	0	—	Strong
Rekhadevi et al. 2007	1	Amount of evaluated cells	Moderate
Cavallo et al. 2007	0	—	Strong
Hongping et al. 2006	1	Blind analysis	Moderate
Hongping et al. 2005	1	Blind analysis	Moderate
Cavallo et al. 2005	1	Blind analysis	Moderate
Yang et al. 2002	1	Blind analysis	Moderate
Hessel et al. 2001	0	—	Strong
Maluf and Erdtmann 2000	1	Blind analysis	Moderate
Pilger et al. 2000	0	—	Strong
Kašuba et al. 1999	1	Blind analysis	Moderate
Fucic et al. 1998	1	Amount of evaluated cells	Moderate
Kevekordes et al. 1998	1	Blind analysis	Moderate
Machado‐Santelli et al. 1994	2	Blind analysis and amount of evaluated cells	Weak
Anwar et al. 1994	0	—	Strong

## 4 Discussion

This systematic review aimed to evaluate the genotoxic effects through the micronucleus assay of occupational exposure to antineoplastic drugs among healthcare professionals. The findings of this review demonstrate a consistent pattern of genotoxic effects associated with exposure to antineoplastic agents. Among the 28 studies included, the vast majority reported increased frequencies of micronuclei in exposed individuals compared to control groups. Notably, micronucleus formation as an endpoint underscores its sensitivity as a biomarker for genotoxicity in occupational biomonitoring, as validated by Bonassi et al. (2011), who demonstrated that increased micronucleus frequency reliably predicts cancer risk and reflects chromosomal damage in exposed human populations.

Conversely, cytotoxicity was less frequently assessed, appearing in only a minority of studies. When evaluated, indicators such as cell viability, apoptosis, and necrosis yielded inconsistent findings. This may be due to methodological limitations or variability in the parameters chosen for cytotoxicity detection. Nonetheless, the presence of cytotoxicity in some cohorts reinforces the biological impact of chronic exposure to cytotoxic agents, especially among pharmacists and nurses handling high volumes of chemotherapy drugs without adequate protective measures.

Among the 28 studies reviewed, only three included the micronucleus assay in oral mucosal cells. Notably, all three reported a significant increase in micronuclei frequency in oral cells, even in cases where no genotoxic alterations were detected in peripheral blood lymphocytes. This finding highlights the potential sensitivity of the oral mucosa as a biomarker for occupational exposure to antineoplastic agents. Despite its diagnostic value and the fact that it is a noninvasive collection method, the use of oral cell assays remains underexplored in the current literature within the field (Thomas et al. 2009).

Furthermore, it is important to highlight that a minimum number of cells is required to ensure the reliability of micronucleus assay results. A total of 1000 cells per subject for peripheral blood lymphocytes and 2000 for oral mucosa cells, as recommended by OECD guidelines (OECD 2016). Among the studies reviewed, this criterion was met or exceeded in nearly all cases that assessed peripheral blood, reinforcing the methodological quality of the findings (only one study analyzing blood samples failed to reach the recommended cell count threshold).

As for oral cell evaluation, two out of the three studies that assessed this tissue failed to reach the recommended threshold of 2000 cells per individual (Bonassi et al. 2011). Despite the reduced number of cells analyzed, both studies still reported an increased frequency of micronuclei, reinforcing the high sensitivity of the oral mucosa as a biomarker site for genotoxic effects. This finding aligns with prior evidence suggesting that epithelial tissues, particularly those with high cell turnover like the oral mucosa, are effective indicators in biomonitoring protocols (Bonassi et al. 2011; Ceppi et al. 2009).

It is important to stress that the genotoxic effects observed were often more pronounced in female participants, as reported in several studies, suggesting that biological sex may modulate susceptibility to genotoxic effects. Although the underlying mechanisms remain unclear and warrant further investigation, sex‐related differences in DNA repair capacity and hormonal influences have been proposed (Hartmann and Speit 2014). Additionally, duration and intensity of exposure were critical factors, with professionals having longer exposure histories, often exceeding 5–10 years, consistently exhibiting higher rates of genotoxic damage, reinforcing the dose–response relationship seen in occupational genotoxicology (Bolognesi 2003).

This variability in genotoxicity induced by antineoplastic drugs may be primarily attributed to genetic polymorphisms that influence DNA repair mechanisms, oxidative stress responses, and apoptosis pathways. For this reason, understanding a genetic component in the variability of population responses to chemicals could be highly valuable for determining individual doses in chemotherapy and selecting individuals for occupations involving chronic exposure (Sivadas et al. 2025). Furthermore, lifestyle factors, such as smoking, can exacerbate the genotoxic effects by generating additional oxidative stress, further complicating the relationship between antineoplastic drugs and genetic damage (Katoh and Katoh 2009). In this study, only a few studies evaluated polymorphisms in DNA repair genes (e.g., XRCC1, XRCC3, hOGG1). While no consistent associations were observed, the inclusion of genetic variables represents an important direction for future research, particularly in identifying subgroups of workers at elevated risk.

The quality of the studies conducted is an important consideration when evaluating the risks of chemotherapy drug exposure. Small sample sizes, lack of control groups, and failure to account for confounding factors can affect the reliability of findings (Connor et al. 2005; Villarini et al. 2016). These factors must be carefully considered when interpreting the results of these studies. Despite the variation in study designs and geographical contexts, a notable strength of this review lies in the overall quality of the included studies. According to our quality assessment, all studies, except one (27 out of the 28), were classified as moderate or strong, reflecting solid methodological rigor. This enhances the credibility of the observed associations and reinforces the urgency of mitigating occupational exposure to these hazardous substances.

Nevertheless, some limitations must be acknowledged. A minority of studies failed to perform blind analyses or fully adjust for confounders such as age, sex, smoking status, and use of personal protective equipment. In addition, cytotoxicity was often overlooked, and dosage or drug concentrations were rarely quantified, impeding a detailed dose–response analysis. A further limitation is the lack of segregation between different chemotherapeutic agents, because each drug's distinct mechanism of action complicates isolating their specific genotoxic effects. Further studies could focus on this segregation.

From a public health perspective, these findings have significant implications. Antineoplastic drugs are inherently clastogenic and aneugenic, designed to target rapidly dividing cancer cells (De Flora 2000). However, their handling by healthcare workers without rigorous safety protocols results in unintended genotoxic consequences. The accumulation of chromosomal damage over time may elevate long‐term cancer risk, underscoring the need for systematic biomonitoring, mandatory use of PPE, proper ventilation systems, and ongoing training on safe handling practices in hospitals and pharmacies (Sessink and Bos 1999).

In conclusion, this systematic review provides robust evidence that occupational exposure to antineoplastic agents is associated with increased genotoxicity in healthcare professionals, particularly nurses and pharmacists, increasing the cancer risk. Therefore, the micronucleus assay is a suitable biomarker for biomonitoring these professionals. Given the high quality of the majority of included studies, these results offer a reliable basis for improving workplace safety regulations, expanding surveillance programs, and promoting institutional awareness regarding the hidden risks of handling antineoplastic drugs.

## Author Contributions

Study design: Thiago Guedes Pinto, Lorrany da Silva Avanci, Daniel Vitor de Souza, Gabriel Carvalhal de Aguiar, and Daniel Araki Ribeiro. Data search: Thiago Guedes Pinto, Daniel Vitor de Souza, and Daniel Araki Ribeiro. Data analysis: Thiago Guedes Pinto, Lorrany da Silva Avanci, Daniel Vitor de Souza, Gabriel Carvalhal de Aguiar, Patricia Ramos Cury, Ana Claudia Muniz Renno, and Daniel Araki Ribeiro. Writing the paper: Thiago Guedes Pinto, Lorrany da Silva Avanci, Daniel Vitor de Souza, Gabriel Carvalhal de Aguiar, Patricia Ramos Cury, Ana Claudia Muniz Renno, and Daniel Araki Ribeiro.

## Conflicts of Interest

The authors declare no conflicts of interest.

## Data Availability

Data sharing not applicable to this article as no datasets were generated or analysed during the current study.
